# *Roseburia* Abundance Associates With Severity, Evolution and Outcome of Acute Ischemic Stroke

**DOI:** 10.3389/fcimb.2021.669322

**Published:** 2021-10-19

**Authors:** Mengmeng Gu, Nihong Chen, Huanhuan Sun, Zhongyuan Li, Xiangliang Chen, Junshan Zhou, Yingdong Zhang

**Affiliations:** ^1^ Department of Neurology, Nanjing First Hospital, Nanjing Medical University, Nanjing, China; ^2^ Department of Neurology, Nanjing Yuhua Hospital, Yuhua Branch of Nanjing First Hospital, Nanjing, China

**Keywords:** gut microbiota, *Roseburia*, minor stroke, fasting glucose, prognosis

## Abstract

Stroke induces disorder of gut microbiota, however, whether this disorder differs according to stroke severity and its role in the evolution and outcome of stroke is currently unknown. Here we explored the composition and structure of fecal microbiome based on 68 acute ischemic stroke patients presenting with minor symptoms (admission National Institute of Health Stroke Scale (NIHSS) ≤ 3) and 67 patients with non-minor stroke (admission NIHSS 4-34) using high-throughput Illumina sequencing of the 16S rRNA. There was no significant difference in α-diversity indices, but the principal coordinate analysis of the microbiota indicated clear separation of the two groups. The significantly enriched butyrate-producing genus *Roseburia* in the minor stroke group was negatively correlated with fasting glucose, while the *Erysipelotrichaceae incertae sedis* abundant in non-minor stroke patients was positively correlated with stress hyperglycemia (i.e. fasting glucose/glycated hemoglobin ratio). Moreover, the relative abundance of genus *Roseburia* was also significantly associated with the dynamic changes of NIHSS score, as well as short-term and long-term functional outcomes. Our results suggested that stroke affects microbiota composition in a manner differentiated by stroke severity, and the enrichment of genus *Roseburia* may play a protective role in stroke evolution and outcome. Our findings strengthen the relevance of specific taxa for stroke severity that might allow targeted therapy in acute ischemic stroke.

## Introduction

Ischemic stroke is a major cause of disability and mortality worldwide, most prominently in adults older than 50 years (Diseases and Injuries, 2020). The severity of neurological deficit is a crucial predictor of clinical outcomes of stroke (Rost et al., 2016; Wouters et al., 2018). Compared with patients experiencing a minor stroke, patients with severe stroke have a higher disability rate (Ferro et al., 2016; Farzadfard et al., 2019) and are more likely to have recurrent vascular events (Park and Ovbiagele, 2016; Asberg et al., 2018). Severe stroke usually means greater irreversible neurological deficit after local brain tissue injury. On the other hand, severe stroke may lead to greater systemic inflammatory response, thus aggravating the symptoms and prognosis of patients (Okar et al., 2020). Accordingly, a better understanding of the factors involved after the onset of stroke in view of different severity and their role in stroke severity trajectory is helpful to predict stroke prognosis and formulate new prevention and treatment strategies. In the context of post-stroke modifiable factors, gut microbiota has emerged as a promising target for shaping a wide variety of factors that will subsequently affect stroke severity and disease progression (Prame Kumar and Wong, 2020).

Gut microbiota interacts with the brain following an ischemic stroke through the bidirectional communication axis known as the microbiota-gut-brain axis (Cryan et al., 2020). Previous studies have shown that gut microbiota not only contributes to the risk factors of stroke such as hypertension (Silveira-Nunes et al., 2020), obesity (Thaiss, 2018), atherosclerosis (Liu and Dai, 2020), glucose and lipid metabolism (Morrison and Preston, 2016; Luck et al., 2019; Martin et al., 2019), but might also be a direct risk factor for stroke (Zeng et al., 2019; Tan et al., 2020). Moreover, the modulation of gut microbiota could have a positive impact on the progression and outcome of ischemic stroke (Spychala et al., 2018). Aside from the bottom-up influences, stroke triggers downstream effects including increased gastrointestinal permeability and dysbiosis of the gut microbiota, mainly manifested by the reduction of short-chain fatty acids (SCFAs)-producing bacteria (Meddings and Swain, 2000; Li et al., 2019; Tan et al., 2021). However, uncertainties remained about the alterations of gut microbiota in different stroke severity and the specific bacterial taxa involved in the dynamic course of neurological deficits due to ischemic stroke.

The aims of this study were to (1) investigate differences in the composition and structure of gut microbiota between hospitalized ischemic stroke patients with different admission severity; (2) explore the correlation between stroke-induced alterations of gut microbiota and biochemical profiles such as glucose and lipids; (3) evaluate the severity-based discriminative taxa in relation to the evolution of subsequent stroke severity, the short-term and long-term functional outcomes.

## Materials and Methods

### Study Participants

This is a prospective observational cohort study conducted in Nanjing First Hospital. Patients with acute ischemic stroke were consecutively recruited from May 2018 to June 2019 with the following inclusion criteria: 1) aged 50 years or older; 2) local residents for over 6 months; 3) Magnetic Resonance Imaging (MRI)-confirmed ischemic stroke in the anterior circulation within 3 days of symptom onset; and 4) signed written informed consents. Exclusion criteria included: 1) cerebral hemorrhagic stroke; 2) a history of chronic inflammatory or immune diseases (e.g., rheumatoid arthritis, systemic lupus erythematosus, or inflammatory bowel disease); 3) a history of severe liver or kidney dysfunction, hematological diseases, and malignancies; 4) administration of probiotics, antibiotics, corticosteroids or immunosuppressants within the past 1 months; and 5) insufficient collection of fecal or blood samples.

The study was approved by the Ethical Review Board of Nanjing First Hospital (Nanjing, China). The patients provided their written informed consent to participate in this study.

### Baseline Characteristics and Sample Collection

We collected demographic information and medical histories from all participants by face-to-face interview. The etiology of ischemic stroke was classified by the Trial of Org 10172 in Acute Stroke Treatment (TOAST) criteria. Large artery atherosclerosis refers to the stroke caused by significant (>50%) atherosclerotic stenosis or occlusion of a major brain artery or branch cortical artery (Adams et al., 1993). Small artery occlusion refers to a recent infarction in the territory of one perforating arteriole, which should be less than 20 mm in its maximum diameter in the axial plane (Wardlaw et al., 2013). Biochemical parameters including serum levels of total cholesterol (TC), high-density lipoprotein cholesterol (HDL), low-density lipoprotein cholesterol (LDL), fasting glucose, glycated hemoglobin, blood urea nitrogen (BUN), serum creatinine (Scr) and uric acid (UA) were collected after overnight fasting within 24 hours of admission and measured at the hospital central laboratory with laboratory staff blinded to clinical data. Stress hyperglycemia (SHG) was also included as a better biomarker of critical illness than absolute hyperglycemia (Roberts et al., 2015). It was calculated using the following formula: fasting glucose/glycated hemoglobin ratio. Stroke severity was assessed by experienced neurologists (H.S and Z.L) on admission using the National Institute of Health Stroke Scale (NIHSS) score and retested at 24 hours, 3 days and 7 days. Patients were divided into two groups: minor stroke, who had admission NIHSS score ≤ 3 (Wang et al., 2013), and non-minor stroke with admission NIHSS score > 3.

Sterile fecal containers and instructions were distributed to each study participant on admission. Approximately 2 g of fresh fecal samples were collected from each participant within 24 hours after admission and immediately (within 1 hour) stored at -80°C until analysis.

### Functional Outcomes

Functional outcomes were quantified using the modified Rankin scale (mRS) score at 30 days and 1 year through routine telephone interview (M.G, H.S and Z.L). Poor functional outcome was defined as mRS score > 2.

### DNA Extraction and High Throughput Sequencing

DNA extraction and sequencing were supported by the Shanghai Genesky Biotechnology Company (Shanghai, China) not knowing group assignment. According to the instructions, fecal genomic DNA was extracted from the fecal samples using the QIAamp^®^ DNA Stool Mini Kit (Qiagen, Hilden, Germany). The V3-V4 hypervariable regions of the bacterial 16S rRNA gene were amplified by polymerase chain reaction (PCR) with the forward primer (5’-CCTACGGGNGGCWGCAG-3’) and the reverse primer (5’-GACTACHVGGGTATCTAATCC-3’) (Meng et al., 2021). Each sample was independently amplified by three repeated PCR experiments. The PCR products were checked by agarose gel electrophoresis, and the products from the same sample were pooled. The pooled PCR product was used as a template, and the index PCR was performed by using index primers for adding the Illumina index to the library. The amplification products were checked using gel electrophoresis and were purified using the Agencourt AMPure XP Kit (Beckman Coulter, CA, USA). The purified products were indexed in the 16S V3-V4 library. The library quality was assessed on the Qubit@2.0 Fluorometer (Thermo Scientific, USA) and Agilent Bioanalyzer 2100 systems (USA). High throughput sequencing was performed on the Illumina Miseq platform using the 2×250 bp paired-end read protocol.

### Bioinformatics and Statistical Analysis

The raw reads were quality filtered and merged with the following criteria: (1) truncation of the raw reads at any site with an average quality score < 20, removal of reads contaminated by adapter and further removal of reads having less than 100 bp by TrimGalore; (2) the paired end reads were merged to tags by Fast Length Adjustment of Short reads (FLASH, v1.2.11); (3) removal of reads with ambiguous bases (N base) and reads with more than 6 bp of homopolymer by Mothur; (4) removal of reads with low complexity to obtain clean reads for further bioinformatics analysis. The remaining unique reads were clustered into operational taxonomic units (OTUs) by UPARSE with a 97% similarity cutoff. All OTUs were classified based on Ribosomal Database Project (RDP) Release 9 by Mothur. Within-individual (α) diversity (including observed species, Chao 1, ACE, Shannon, Simpson, and Coverage index) was used to measure the richness or evenness of taxa within each sample, and was analyzed by Mothur. Between-individual (β) diversity was provided for comparison of the taxonomic profiles between microbial communities. Unweighted UniFrac is a qualitative measure of the distance between microbial communities by calculating the fraction of the branch length in a phylogenetic tree (Lozupone and Knight, 2005). Weighted UniFrac further weights the branches of a phylogenetic tree based on the abundance of information, which is a quantitative measure (Chang et al., 2011). Unweighted and weighted UniFrac principal coordinate analysis (PCoA) based on OTUs were performed by R version 3.4.3 (Vegan package). Permutational multivariate analysis of variance (PERMANOVA; Adonis function) was carried out to examine whether there were statistical differences in bacterial community composition (β-diversity) between groups. Metastats analysis and linear discriminant analysis (LDA) effect size (LEfSe) were used to determine the significantly discriminative taxa between groups (Segata et al., 2011). Bacteria with significant differences (absolute value of logarithmic LDA score > 2) between the two groups were plotted on taxonomic bar plots. We also used BugBase to predict potential microbiome phenotypes, including aerobic, anaerobic, containing mobile elements, facultatively anaerobic, biofilm forming, gram-negative, gram-positive, potentially pathogenic, and stress tolerant (Ward et al., 2017).

All statistical analyses were performed with R version 3.4.3 (R Development Core Team, Vienna, Austria). Continuous variables were expressed as median [interquartile range (IQR)] or mean ± standard deviation (SD) and compared with Wilcoxon rank sum test or student *t* test when appropriate. Categorical variables were expressed as number (percentage) and compared by Pearson’s chi-square test. A box-plot outlier is detected as any observation that falls below the lower fence (LF) [LF = Q1-1.5*IQR] or above the upper fence (UF) [UF = Q3+1.5*IQR], where Q1, Q3 and IQR are the first quartile, third quartile and interquartile range of the data, respectively (Chervoneva et al., 2006). The missing values of TC (1.5%), HDL (1.5%), LDL (1.5%), fasting glucose (3.7%), glycated hemoglobin ratio (3.7%), and UA (5.9%) were interpolated with the median. Propensity score-matched (PSM) analysis was used to obtain matched pairs of samples from the minor stroke group and the non-minor stroke group. In the PSM algorithm, the corresponding propensity score of the grouping variable (minor or non-minor) was calculated for each patient with a 1:1 nearest-neighbor matching algorithm with a caliper width of 0.2 of the propensity score, with age, sex, and coronary heart disease (CHD) as covariates. Spearman’s rank correlation coefficient was used to explore the correlation of different genera with biochemical parameters, NIHSS scores obtained at different timepoints and functional outcomes. We used linear mixed-effects models with random intercepts and slopes to test whether the relative abundance of discriminative taxa (e.g., genus *Roseburia*) or *Firmicutes* to *Bacteroidetes* ratio (F/B ratio) or gram-negative/gram-positive ratio account for the evolution of NIHSS scores through the first 7 days of hospitalization. Since the NIHSS score was highly skewed, the natural logarithm transformation [ln (NIHSS + 1)] was applied. Grand-mean centering for continuous covariates with meaningless 0 values (such as age) was performed. Multivariable logistic regression analyses were also used to evaluate the associations between the relative abundance of discriminative taxa and functional outcomes at 30 days and 1 year. The resulting *p* values were adjusted using the Benjamini-Hochberg false discovery rate (FDR) correction. Two-sided *p* value < 0.05 was considered significant.

## Results

### Baseline Characteristics of Patients

A total of 732 patients were screened and 135 were recruited according to the inclusion and exclusion criteria. The flowchart for the selection is presented in **Figure S1**. A total of 46 (34.1%) patients received intravenous thrombolysis. The NIHSS score of non-minor stroke group ranged from 4 to 34. Patients with minor stroke (n = 68) were younger than those with non-minor stroke (n = 67) (*t* test, *p* = 0.001). CHD was more common in the non-minor stroke patients (*χ^2^* test, *p* = 0.008), whereas small artery occlusion was more common in the minor stroke patients (*χ^2^* test, *p* < 0.001; **Table 1**). After PSM, 48 patients in the minor stroke group were matched to 48 patients in the non-minor stroke group, and no significant difference was observed in baseline characteristics between the two groups (**Table 1**).

**Table 1:** Baseline characteristics of patients before PSM and after PSM.

Baseline characteristics	Before PSM			After PSM		
	Minor stroke (n = 68)	Non-minor stroke (n = 67)	P value	Minor stroke (N = 48)	Non-minor stroke (N = 48)	P value
Age, mean ± SD, y	65.0 ± 9.2	71.0 ± 10.4	**0.001**	66.8 ± 9.1	67.7 ± 9.3	0.625
Male, n (%)	48 (70.6)	43 (64.2)	0.427	36 (75.0)	34 (70.8)	0.646
BMI, mean ± SD, kg/m^2^*	23.9 ± 3.5	24.7 ± 3.7	0.196	24.2 ± 3.6	25.2 ± 3.4	0.164
Hypertension, n (%)	55 (80.9)	56 (83.6)	0.682	39 (81.3)	39 (81.3)	1.000
Diabetes mellitus, n (%)	27 (39.7)	23 (34.3)	0.518	20 (41.7)	15 (31.3)	0.289
Dyslipidemia, n (%)	42 (61.8)	39 (58.2)	0.673	27 (56.3)	29 (60.4)	0.679
Coronary heart disease, n (%)	2 (2.9)	11 (16.4)	**0.008**	2 (4.2)	2 (4.2)	1.000
Smoking, n (%)	38 (55.9)	39 (58.2)	0.785	27 (56.3)	31 (64.6)	0.404
Drinking, n (%)	25 (36.8)	29 (43.3)	0.440	17 (35.4)	22 (45.8)	0.299
TOAST, n (%)			**<0.001**			0.054
LAA	27 (39.7)	26 (38.8)		22 (45.8)	23 (47.9)	
SAO	32 (47.1)	13 (19.4)		20 (41.7)	11 (22.9)	
Others	9 (13.2)	28 (41.8)		6 (12.5)	14 (29.2)	

*For 2 patients, BMI was not available.

Bold value: p value with statistical significance.

PSM, propensity score-matched analysis; SD, standard deviation; BMI, body mass index; TOAST, Trial of Org 10172 in Acute Stroke Treatment; LAA, large artery atherosclerosis; SAO, small artery occlusion.

### The Structure of the Gut Microbiota in Patients

**Figure 1:**
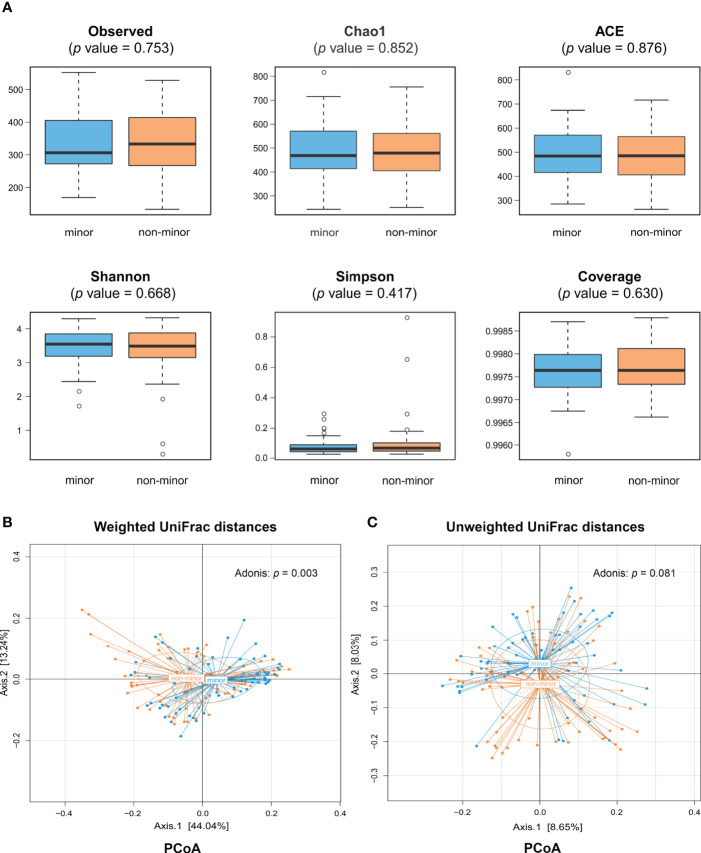
Comparison of gut microbiota diversity between minor stroke patients and non-minor stroke patients. **(A)** Within-individual (α) diversity including observed species, Chao 1 and ACE, Shannon index, Simpson index and coverage index in minor stroke patients (blue) and non-minor stroke patients (orange). Boxes represent the interquartile ranges, lines inside the boxes denote medians, and circles are outliers. Between-individual (β) diversity, including principal coordinate analysis (PCoA) based on weighted UniFrac distances **(B)** and unweighted UniFrac distances **(C)**, was tested by Adonis. The blue circles represent samples of minor stroke patients and orange circles represent samples of non-minor stroke patients. PCoA, principle coordinate analysis.

A total of 22,689,893 high-quality reads (mean per subject, 168,073; range, 62,541-992,110) were obtained and analyzed. Of these, 89.0% were clean reads. Reads were clustered into 2,600 OTUs at 97% identity. There was no significant difference in the α-diversity indices between minor and non-minor stroke, including the richness (observed species, Chao 1, and ACE) and diversity (Shannon index, Simpson index, and Coverage index) of microbial communities, whether before or after PSM (Wilcoxon rank sum test, *p* > 0.05) (**Figures 1A** and **S2A**).

The results of PCoA showed that the microbial structure of patients with minor stroke was different from that of patients with non-minor stroke in weighted UniFrac distances (Adonis, *p* = 0.003; **Figure 1B**). Adonis using an unweighted UniFrac distance, however, could not detect community composition difference between the two groups (Adonis, *p* = 0.081; **Figure 1C**). Nevertheless, after PSM, we found significant differences in microbial communities between groups, which was consistent with the Adonis results of weighted UniFrac distance (**Figures S2B, C**).

### Difference in the Microbial Composition at Different Levels

**Figure 2:**
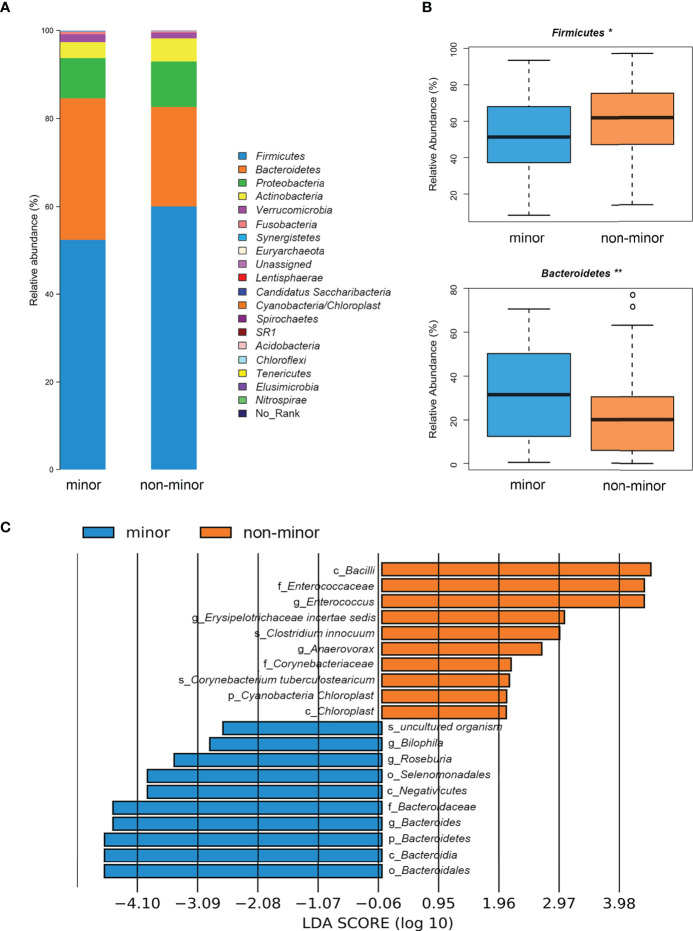
Difference of microbial composition between minor stroke patients and non-minor stroke patients. **(A)** Taxonomic summary of the gut microbiota of minor stroke patients and non-minor stroke patients at the phylum level. **(B)** Bacteria with significant differences between minor stroke patients (blue) and non-minor stroke patients (orange) at the phylum level (Metastats analysis). Boxes represent the interquartile ranges, lines inside the boxes denote medians, and circles are outliers. **(C)** Significantly discriminative taxa between the minor stroke patients (blue) and non-minor stroke patients (orange) determined by linear discriminant analysis effect size (LDA effect size). *p < 0.05, **p < 0.01.

We evaluated the differences in the composition of gut microbial community. **Figure 2A** showed that most of the gut bacteria detected fall into 3 phyla: *Firmicutes*, *Bacteroidetes*, and *Proteobacteria*. Compared with the minor stroke group, the relative abundance of phylum *Firmicutes* was significantly higher in the non-minor stroke group, while the beneficial phylum *Bacteroidetes* was relatively less abundant (**Figure 2B**). After PSM, the relative abundance of phyla *Firmicutes* and *Bacteroidetes* were still significantly different between the two groups (**Figure S3**). Furthermore, the F/B ratio of the non-minor stroke group was significantly higher than that of the minor stroke group (Wilcoxon rank sum test, *p* = 0.001, **Table 2**).

**Table 2:** Metabolic parameters and microbial composition of patients.

	Minor stroke (N = 68)	Non-minor stroke (N = 67)	P value
TC, median (IQR), mmol/L	4.32 (3.52-5.07)	4.37 (3.76-4.81)	0.948
HDL, median (IQR), mmol/L	1.08 (0.95-1.20)	1.08 (0.92-1.21)	0.672
LDL, median (IQR), mmol/L	2.48 (1.86-3.17)	2.60 (2.06-3.15)	0.572
Fasting glucose, median (IQR), mmol/L	5.07 (4.41-6.47)	5.39 (4.75-6.90)	0.075
SHG, median (IQR), mmol/L/%	0.87 (0.75-0.96)	0.93 (0.80-1.10)	0.017
BUN, median (IQR), mmol/L	5.62 (4.43-6.66)	5.40 (4.50-6.70)	0.610
Scr, median (IQR), μmoI/L	71.7 (58.4-84.4)	69.9 (59.0-86.0)	0.893
UA, median (IQR), μmoI/L	294.5 (241.8-360.3)	303.0 (219.0-358.5)	0.380
F/B ratio, median (IQR)	1.73 (0.76-5.38)	3.27 (1.48-14.89)	**0.001**

The number of missing values of TC, HDL, LDL, fasting glucose, SHG and UA were 2, 2, 2, 2, 5, 10 and 8 respectively.

Bold value: p value with statistical significance.

TC, total cholesterol; IQR, interquartile range; HDL, high-density lipoprotein cholesterol; LDL, low-density lipoprotein cholesterol; SHG, stress hyperglycemia (fasting glucose/glycated hemoglobin ratio); BUN, blood urea nitrogen; Scr, serum creatinine; UA, uric acid; F/B ratio, Firmicutes to Bacteroidetes ratio.

LEfSe analysis and LDA score were used to further identify the microbial species with significant differences between the two groups from phylum to the genus levels. At the genus level, *Bilophila*, *Roseburia*, and *Bacteroides* were significantly enriched in the minor stroke group, while *Erysipelotrichaceae incertae sedis*, *Enterococcus*, and *Anaerovorax* were more abundant in the non-minor stroke group (**Figure 2C**). After PSM, the genus *Bacteroides* and *Roseburia* still increased significantly in patients with minor stroke (**Figure S4**).

**Figure 3:**
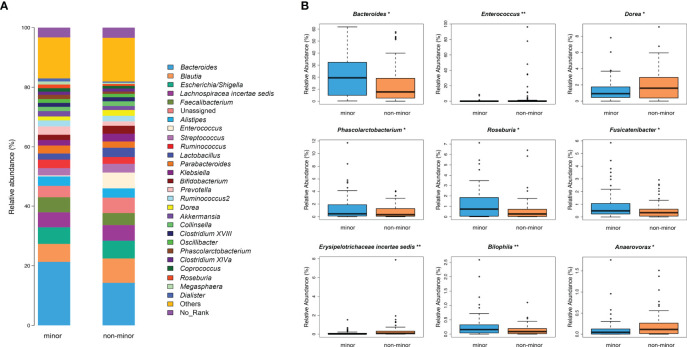
Genus-level difference of microbial composition between minor stroke patients and non-minor stroke patients. **(A)** Taxonomic summary of the gut microbiota of minor stroke patients and non-minor stroke patients at the genus level. **(B)** Bacteria with significant differences between minor stroke patients (blue) and non-minor stroke patients (orange) at the genus level (Metastats analysis). Boxes represent the interquartile ranges, lines inside the boxes denote medians, and circles are outliers. *p < 0.05, **p < 0.01.

The composition of bacterial communities at the genus level is shown in **Figure 3A**. Metastats analysis showed significantly more abundant SCFAs-producing bacteria (i.e., *Roseburia*, *Bacteroides* and *Phascolarctobacterium*) in the minor stroke group than those in the non-minor stroke group. While genus *Dorea*, *Erysipelotrichaceae incertae sedis*, *Enterococcus*, and *Anaerovorax* were more abundant in patients with non-minor stroke (**Figure 3B**).

### Potential Microbiome Phenotypes

Using BugBase, nine potential phenotypes were predicted (**Table 3**). Compared with the non-minor stroke group, the minor stroke group exhibited significantly lower levels of the relative abundances of the gram-positive (*t* test, *p* = 0.020) and mobile elements containing (*t* test, *p* = 0.010) phenotypes, whilst higher levels of the relative abundances of the gram-negative (*t* test, *p* = 0.023) and potentially pathogenic (*t* test, *p* = 0.008) phenotypes. There was no significant difference in the ratio of gram-negative/gram-positive between the two groups (*t* test, *p* = 0.059, **Table 3**).

**Table 3:** Relative abundances of nine potential phenotypes predicted by BugBase in two groups.

Phenotypes (proportion)	Minor stroke (N = 68)	Non-minor stroke (N = 67)	P value
Aerobic, mean ± SD	0.01 ± 0.02	0.01 ± 0.02	0.824
Anaerobic, mean ± SD	0.26 ± 0.03	0.24 ± 0.06	0.103
Contains mobile elements, mean ± SD	0.25 ± 0.04	0.27 ± 0.05	**0.010**
Facultatively anaerobic, mean ± SD	0.02 ± 0.02	0.02 ± 0.02	0.271
Forms biofilms, mean ± SD	0.03 ± 0.02	0.04 ± 0.03	0.234
Gram-negative, mean ± SD	0.09 ± 0.05	0.07 ± 0.05	**0.023**
Gram-positive, mean ± SD	0.21 ± 0.06	0.23 ± 0.06	**0.020**
Potentially pathogenic, mean ± SD	0.12 ± 0.05	0.10 ± 0.05	**0.008**
Stress tolerant, mean ± SD	0.01 ± 0.01	0.01 ± 0.02	0.543
Gram-negative/gram-positive ratio, mean ± SD	0.58 ± 0.63	0.40 ± 0.48	0.059

Bold value: p value with statistical significance. SD, standard deviation.

### Correlation Analysis Between Gut Bacteria and Biochemical Parameters

There were no statistically significant differences in TC, HDL, LDL, fasting glucose, BUN, Scr and UA levels between the two groups (**Table 2**). The levels of SHG and F/B ratio in the non-minor stroke group were significantly higher than that in the minor stroke group (**Table 2**).

**Figure 4:**
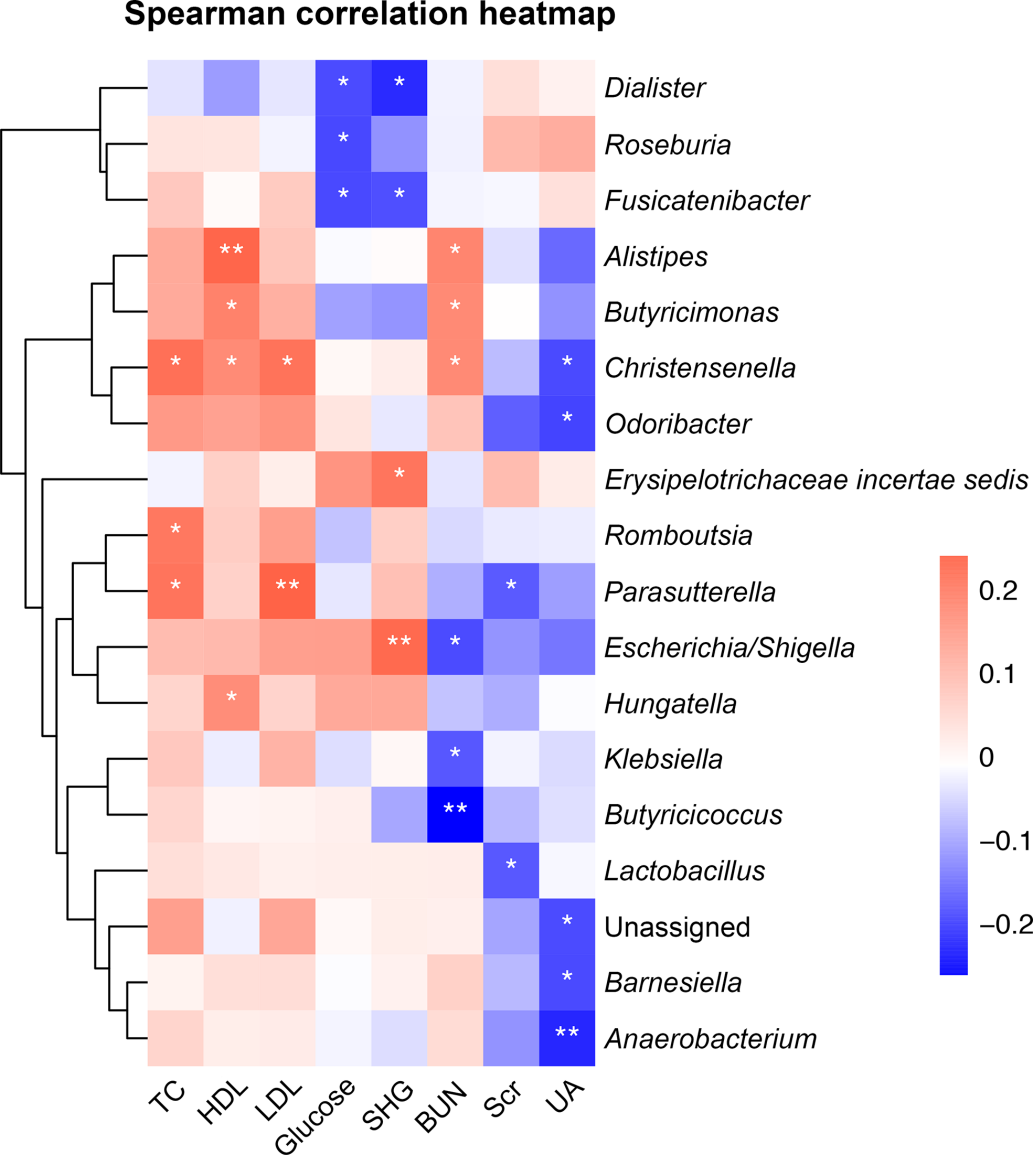
Heatmap of Spearman correlation analysis between gut microbiota and biochemical parameters. *p < 0.05, **p < 0.01. TC, total cholesterol; HDL, high-density lipoprotein cholesterol; LDL, low-density lipoprotein cholesterol; SHG, stress hyperglycemia; BUN, blood urea nitrogen; Scr, serum creatinine; UA, uric acid.

We performed correlation analyses between the top-50 common genera (**Table S1**) and biochemical parameters. The results showed that the enriched genus *Erysipelotrichaceae incertae sedis* in the non-minor stroke group was positively correlated with the SHG (Spearman correlation, *r* = 0.21, *p* < 0.05, **Figure 4**). While the enriched genus *Roseburia* in the minor stroke group was negatively correlated with fasting glucose (Spearman correlation, *r* = -0.19, *p* < 0.05, **Figure 4**). Likewise, a similar significantly negative correlation was observed between the abundance of *Roseburia* and fasting glucose after PSM (Spearman correlation, *r* = -0.23, *p* < 0.05, **Figure S5**). Besides, the butyrate-producing genus *Dialister* showed a constantly negative correlation with fasting glucose and SHG, and *Butyricicoccus* with BUN. Genus *Escherichia/Shigella* was positively correlated with SHG and negatively correlated with BUN, and *Parasutterella* was positively correlated with LDL.

### The Dynamic Association Between Gut Microbiota and Stroke Severity

**Figure 5:**
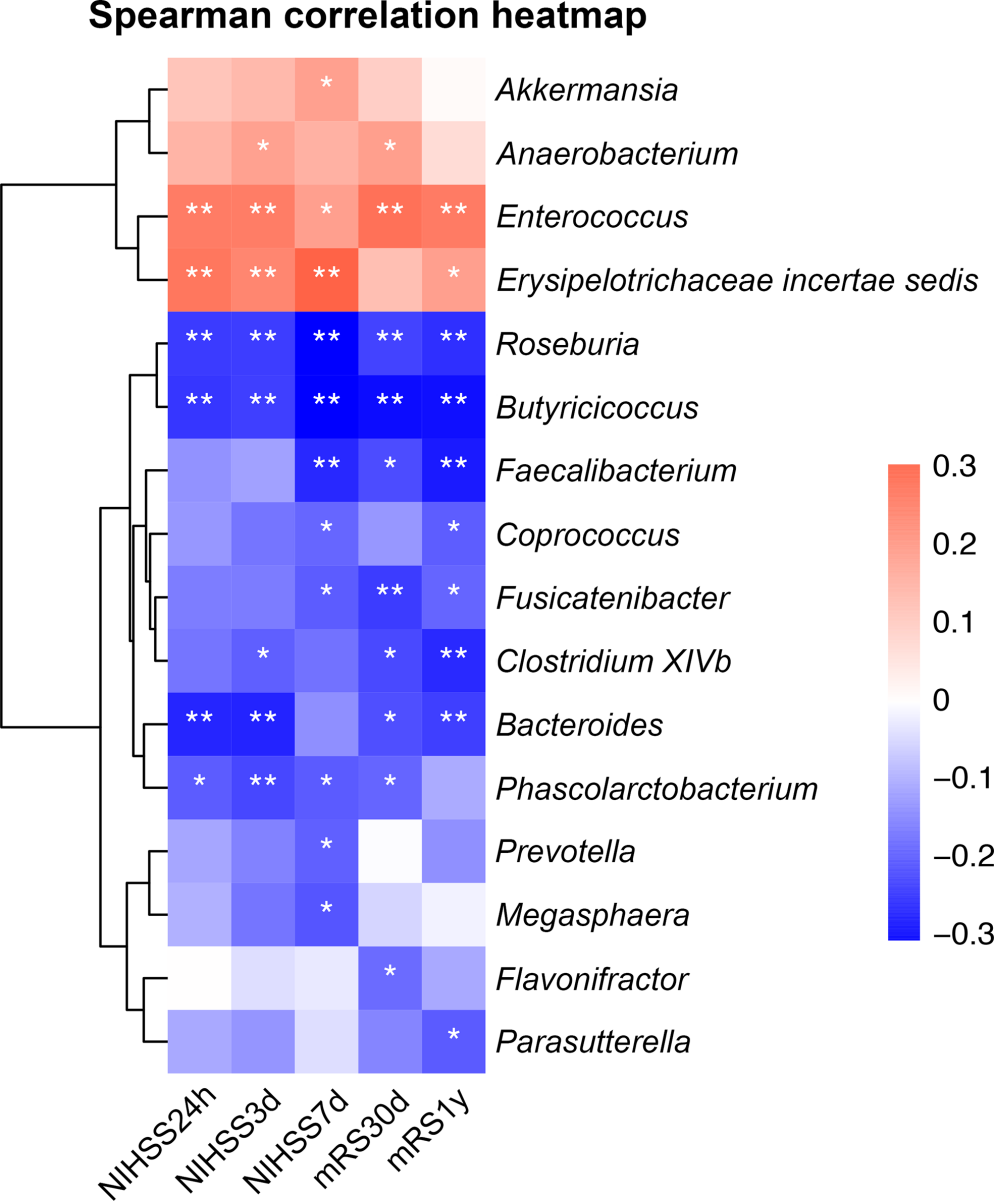
Heatmap of Spearman correlation analysis between gut microbiota and NIHSS score and functional outcomes. *p < 0.05, **p < 0.01. NIHSS, the National Institute of Health Stroke Scale; mRS, modified Rankin scale score.

At 30 days and 1 year, 1 and 5 patients were lost to follow-up, respectively. As shown in **Figure 5**, SCFAs-producing bacterium *Bacteroides* was negatively correlated with 24-hour (Spearman correlation, *r* = -0.26, *p* < 0.01) and 3-day NIHSS score (Spearman correlation, *r* = -0.27, *p* < 0.01), while SCFAs-producing bacteria *Roseburia* and *Butyricicoccus* were negatively correlated with NIHSS at all times (Spearman correlation, *p* < 0.001). Meanwhile, *Erysipelotrichaceae incertae sedis* and *Enterococcus* were significantly positively correlated with NIHSS scores at all times (Spearman correlation, *p* < 0.05). Furthermore, SCFAs-producing bacteria *Faecalibacterium*, *Roseburia* and *Bacteroides* had a negative correlation with the mRS scores at 30 days and 1 year (Spearman correlation, *p* < 0.05), while a positive correlation was noted for *Enterococcus* at both follow-ups (Spearman correlation, *p* < 0.01), and *Erysipelotrichaceae incertae sedis* was correlated with mRS score only at the 1-year follow-up (Spearman correlation, *r* = 0.18, *p* = 0.038).

We chose the significantly discriminative taxa between groups before and after PSM (genus *Bacteroides* and *Roseburia*) to investigate their associations with short-term and long-term prognosis. The results of linear mixed-effects model showed that, the relative abundance of genus *Roseburia* was associated with the ln(NIHSS+1) score (estimate = -13.42, *p* = 0.015). After adjusting for sex, age, CHD, stroke etiology and intravenous thrombolysis, the relationship was still statistically significant (estimate = -10.72, *p* = 0.047). Besides, gram-negative/gram-positive ratio (estimate = -0.28, *p* = 0.034) and the relative abundance of genus *Bacteroides* (estimate = -1.13, *p* = 0.010) were also significantly associated with the ln(NIHSS+1) score. However, F/B ratio (estimate = 0.002, *p* = 0.167) had no correlation with the ln(NIHSS+1) score.

We also used multivariable logistic regression analyses to investigate the associations between the relative abundance of genus *Roseburia* or *Bacteroides* and 30-day and 1-year functional outcomes. Considering that the relative abundance of *Roseburia* is relatively small (median 0.00407, interquartile range: 0.00045-0.01324), we multiplied it by 100 to yield a percentage. After adjusting for sex, age, CHD, stroke etiology and intravenous thrombolysis, the results of multivariable logistic regression analyses showed that the relative abundance of genus *Roseburia* was negatively associated with poor functional outcome at 30 days [odds ratio (OR) 0.50, 95% confidence interval (CI): 0.26-0.96, *p* = 0.036] and 1 year (OR 0.45, 95%CI: 0.22-0.92, *p* = 0.030). However, the relative abundance of genus *Bacteroides* was not associated with poor functional outcomes, whether at 30 days (OR 0.14, 95%CI: 0.01-2.69, *p* = 0.194) or 1 year (OR 0.05, 95%CI: 0.02-1.15, *p* = 0.061). In addition, neither F/B ratio nor gram-negative/gram-positive ratio were associated with short-term and long-term functional outcomes.

## Discussion

This prospective cohort of 135 stroke patients showed that (1) despite the similar α-diversity indices, some gut microbiota genera, like *Roseburia* and *Erysipelotrichaceae incertae sedis*, were distinct between patients with minor and non-minor stroke; (2) *Roseburia* was negatively correlated with fasting glucose and *Erysipelotrichaceae incertae sedis* was positively correlated with SHG; (3) the relative abundance of genus *Roseburia* was significantly associated with the evolution of NIHSS score and short-term and long-term functional outcomes.

Our study is one of the few studies thus far demonstrating the role of gut microbiota in patients with different stroke severity. Yin et al. found that *Bacteroides* were depleted in severe stroke patients compared with mild stroke patients (NIHSS score ≤ 4), whereas *Escherichia/Shigella* were more abundant in severe stroke patients (NIHSS score > 4) (Yin et al., 2015). Consistently, we found that patients with non-minor stroke have lower levels of *Bacteroides* than patients with minor stroke, and the genus *Escherichia/Shigella* was positively correlated to SHG, a marker of disease severity (Pan et al., 2017). In another study, Li et al. reported an increase in *Enterobacter*, *Pyramidobacter*, and *Lachnospiraceae* UCG-001 in mild stroke patients (NIHSS score ≤ 4). Meanwhile, genus *Ruminococcaceae* UCG-002, *Christensenellaceae* R-7 group, *Ruminococcaceae* UCG-005, and *norank Ruminococcaceae* were increased in severe stroke patients (NIHSS score > 4) (Li et al., 2019). However, similar results were not confirmed in our study, this might be attributed to the differences in the definition of minor stroke and the sample size of the study cohort. In the study of Li et al., there were 13 patients with severe stroke and 17 patients with mild stroke. They defined minor stroke as NIHSS score ≤ 4. In addition, the time of enrollment may also account for the discrepancy (Jeon et al., 2020). For instance, they did not define the onset time of stroke and they collected fecal samples within 48 hours of admission.

An important finding of this study is the robust relationship between genus *Roseburia* and stroke severity, evolution and outcome. Genus *Roseburia* is an important butyrate-producing bacterium (Boesmans et al., 2018; Kasahara et al., 2018; Seo et al., 2020). SCFAs, including acetate, propionate, and butyrate, are important bacterial metabolites and have beneficial effects on energy metabolism and intestinal barrier integrity (Kasubuchi et al., 2015). In our study, other SCFAs-producing bacteria such as *Bacteroides* and *Phascolarctobacterium* were also significantly more abundant in the minor stroke group than the non-minor stroke group. Previous studies have confirmed the importance of butyrate-producing bacteria in regulating blood glucose and improving insulin sensitivity (Furet et al., 2010). Compared with patients with normal carbohydrate metabolism, the concentrations of butyrate-producing bacteria in patients with type 2 diabetes were lower (Qin et al., 2012; Karlsson et al., 2013). Transplantation of gut microbiota from lean donors to recipients with metabolic syndrome has been shown to increase levels of butyrate-producing microbiota and improve insulin sensitivity (Vrieze et al., 2012). In our study, the genus *Roseburia* was also negatively correlated with fasting glucose. In addition, previous studies have provided strong evidence for butyrate-producing microbiota against lipid disorders, systemic inflammation, and atherosclerosis (Gozd-Barszczewska et al., 2017; Kasahara et al., 2018; van den Munckhof et al., 2018). SCFAs are also considered as potential mediators of gut microbiota affecting intestinal immune function (Vinolo et al., 2011). The transplantation of SCFA-producers could increase the concentration in gut, brain and plasma, and reduce the neurological deficit and inflammation after stroke in aged mice (Lee et al., 2020). There is also evidence that the microbiota-derived SCFAs have also been shown to modulate poststroke recovery by affecting systemic and brain resident immune cells (Sadler et al., 2020). Thus, transplantation of fecal bacteria rich in SCFAs and supplementation of butyrate might be an effective intervention for poststroke neuroinflammation.

In our study, *Erysipelotrichaceae incertae sedis* was more abundant in patients with non-minor stroke, and was positively correlated with SHG and NIHSS scores. The role of family *Erysipelotrichaceae* in metabolic disorders might account for its potential detrimental effect on stroke severity. Early evidence suggested that species belonging to *Erysipelotrichaceae* would proliferate in diet-induced obese animals (Turnbaugh et al., 2008). The subsequent research observed an increase of *Erysipelotrichaceae* in mice on high-fat or western diet (Fleissner et al., 2010). Nutrition studies further support the effect of dietary fat on the abundance of *Erysipelotrichaceae* (Kaakoush, 2015). Future investigations are needed on the metabolic interactions with *Erysipelotrichaceae* under disease states such as a stroke.

Considering the high disability rate of stroke, it is important to predict the change of stroke severity. As a result of this paper, some bacteria such as *Roseburia*, *Enterococcus*, and *Erysipelotrichaceae incertae sedis* were significantly correlated with the NIHSS score. Besides, the F/B ratio of non-minor stroke patients was higher than that of minor stroke patients, which was consistent with previous reports (Tan et al., 2021). Several previous reports demonstrated that the F/B ratio was associated with obesity, hypertension, diabetes, and atherosclerosis, which are risk factors for stroke. Obese individuals had a significantly higher level of *Firmicutes* and lower level of *Bacteroidetes* compared with lean controls (Ley et al., 2006; Koliada et al., 2017). An increased F/B ratio was observed both in animal and human hypertension (Yang et al., 2015). Demirci et al. reported that the F/B ratio in patients with type 1 diabetes mellitus was significantly lower than in healthy controls (Demirci et al., 2020). Compared with the healthy control group, the F/B ratio of atherosclerotic patients was significantly higher (Emoto et al., 2016). At the same time, studies have shown that a high-fat diet profoundly increases the F/B ratio, resulting in dysregulation of the gut microbiota (Marques et al., 2016). Animal experiments have shown that reducing the F/B ratio of old mice to a level similar to that of young mice could improve the prognosis of stroke (Spychala et al., 2018). These might be the reasons why the F/B ratio predicts the change of stroke severity. However, in this study, after adjusting for covariates, F/B ratio was no longer associated with the evolution of stroke severity. This may be attributed to the older age of patients in the non-minor stroke group. Additional investigations are warranted to further examine the correlations between the F/B ratio and the stroke severity and its possible pathophysiological mechanism.

The findings of our study should be interpreted with caution due to the small sample size and study limitations inherent in any single-center observational analysis. First of all, this is a hospital-based study making it impractical to obtain fecal samples before stroke onset, and the absence of healthy controls makes it different to calibrate the stroke-induced change in the composition of gut microbiota. As such, further studies are needed to explore the effects of the stroke itself on individual gut microbiota. Secondly, we collected the fecal microbiota and measured the biochemical parameters at a single time point, therefore, we could not observe the dynamic changes of the microbiota to test the real-time interaction with these parameters and stroke severity. Thirdly, although we included relatively homogenous participants (i.e. local residents) and excluded those who had used probiotics, antibiotics, corticosteroids, or immunosuppressants in the past month, dietary information and other drugs that may affect gut microbiota were not considered. Another limitation is the lack of characterization of the metabolomics profiles and SCFAs concentrations in the fecal and blood samples. Finally, the observed results might be overstated without the functional correlation or intervention experiments in human or animal models. However, it provides the rationale for large-scale and well-designed studies to explore the prognostic and therapeutic potentials of the genus *Roseburia* in stroke patients.

In summary, our study suggests that acute ischemic stroke patients with different severity have different gut microbiological characteristics. Moreover, the levels of some gut microbiota were related to glucose and lipid metabolism. The relative abundance of genus *Roseburia* could be a predictor of evolution in stroke severity and functional outcomes.

## Data Availability Statement

The datasets presented in this study can be found in online repositories. The names of the repository/repositories and accession number(s) can be found below: FigShare repository (https://figshare.com/), accession number Genome Sequence Archive repository (DOI: 10.6084/m9.figshare.13096223); (https://bigd.big.ac.cn/gsa/browse/CRA004134) (Wang et al., 2017), accession number CRA004134.

## Ethics Statement

The study was approved by the Ethical Review Board of Nanjing First Hospital (Nanjing, China). The patients/participants provided their written informed consent to participate in this study.

## Author Contributions

MG, XC and YZ conceived the study. MG and NC analyzed the data. MG, HS and ZL interpreted the data. MG wrote the manuscript. XC and JZ revised the manuscript. All authors contributed to the article and approved the submitted version.

## Funding

This study was supported by National Natural Science Foundation of China (81701064) and Natural Science Foundation of Jiangsu Province (BK20201117).

## Conflict of Interest

The authors declare that the research was conducted in the absence of any commercial or financial relationships that could be construed as a potential conflict of interest.

## Publisher’s Note

All claims expressed in this article are solely those of the authors and do not necessarily represent those of their affiliated organizations, or those of the publisher, the editors and the reviewers. Any product that may be evaluated in this article, or claim that may be made by its manufacturer, is not guaranteed or endorsed by the publisher.

## Abbreviations

SCFAs, short-chain fatty acids; MRI, Magnetic Resonance Imaging; TOAST, the Trial of Org 10172 in Acute Stroke Treatment; TC, total cholesterol; HDL, high-density lipoprotein cholesterol; LDL, low-density lipoprotein cholesterol; BUN, blood urea nitrogen; Scr, serum creatinine; UA, uric acid; SHG, stress hyperglycemia; NIHSS, National Institute of Health Stroke Scale; mRS, modified Rankin scale; PCR, polymerase chain reaction; OTUs, operational taxonomic units; RDP, Ribosomal Database Project; PCoA, principal coordinate analysis; PERMANOVA, Permutational multivariate analysis of variance; LDA, linear discriminant analysis; LEfSe, linear discriminant analysis effect size; IQR, interquartile range; SD, standard deviation; LF, lower fence; UF, upper fence; PSM, propensity score-matched; CHD, coronary heart disease; F/B ratio, *Firmicutes* to *Bacteroidetes* ratio; FDR, false discovery rate; OR, odds ratio; CI, confidence interval.

## References

[B1] Adams H. P. Jr. Bendixen B. H. Kappelle L. J. Biller J. Love B. B. Gordon D. L.. (1993). Classification of Subtype of Acute Ischemic Stroke. Definitions for Use in a Multicenter Clinical Trial. TOAST. Trial of Org 10172 in Acute Stroke Treatment. Stroke 24, 35–41. doi: 10.1161/01.str.24.1.35 7678184

[B2] Asberg S. Farahmand B. Hasvold P. Johansson S. Appelros P. (2018). Non-Cardioembolic TIA and Ischemic Stroke: Implications of Severity. Acta Neurol. Scand. 138, 369–376. doi: 10.1111/ane.12974 29920644

[B3] Boesmans L. Valles-Colomer M. Wang J. Eeckhaut V. Falony G. Ducatelle R.. (2018). Butyrate Producers as Potential Next-Generation Probiotics: Safety Assessment of the Administration of Butyricicoccus Pullicaecorum to Healthy Volunteers. mSystems 3, e00094–18. doi: 10.1128/mSystems.00094-18 PMC6222043 30417112

[B4] Chang Q. Luan Y. Sun F. (2011). Variance Adjusted Weighted UniFrac: A Powerful Beta Diversity Measure for Comparing Communities Based on Phylogeny. BMC Bioinformatics 12, 118. doi: 10.1186/1471-2105-12-118 21518444 PMC3108311

[B5] Chervoneva I. Hyslop T. Iglewicz B. Johns L. Wolfe H. R. Schulz S.. (2006). Statistical Algorithm for Assuring Similar Efficiency in Standards and Samples for Absolute Quantification by Real-Time Reverse Transcription Polymerase Chain Reaction. Anal. Biochem. 348, 198–208. doi: 10.1016/j.ab.2005.10.042 16336939

[B6] Cryan J. F. O’Riordan K. J. Sandhu K. Peterson V. Dinan T. G. (2020). The Gut Microbiome in Neurological Disorders. Lancet Neurol. 19, 179–194. doi: 10.1016/S1474-4422(19)30356-4 31753762

[B7] Demirci M. Bahar Tokman H. Taner Z. Keskin F. E. Cagatay P. Ozturk Bakar Y.. (2020). Bacteroidetes and Firmicutes Levels in Gut Microbiota and Effects of Hosts TLR2/TLR4 Gene Expression Levels in Adult Type 1 Diabetes Patients in Istanbul, Turkey. J. Diabetes Complicat. 34, 107449. doi: 10.1016/j.jdiacomp.2019.107449 31677982

[B8] Diseases G. B. D. Injuries C. (2020). Global Burden of 369 Diseases and Injuries in 204 Countries and Territories 1990-2019: A Systematic Analysis for the Global Burden of Disease Study 2019. Lancet 396, 1204–1222. doi: 10.1016/S0140-6736(20)30925-9 33069326 PMC7567026

[B9] Emoto T. Yamashita T. Sasaki N. Hirota Y. Hayashi T. So A.. (2016). Analysis of Gut Microbiota in Coronary Artery Disease Patients: A Possible Link Between Gut Microbiota and Coronary Artery Disease. J. Atheroscler. Thromb. 23, 908–921. doi: 10.5551/jat.32672 26947598 PMC7399299

[B10] Farzadfard M. T. Sheikh Andalibi M. S. Thrift A. G. Morovatdar N. Stranges S. Amiri A.. (2019). Long-Term Disability After Stroke in Iran: Evidence From the Mashhad Stroke Incidence Study. Int. J. Stroke 14, 44–47. doi: 10.1177/1747493018789839 30117788

[B11] Ferro J. M. Caeiro L. Figueira M. L. (2016). Neuropsychiatric Sequelae of Stroke. Nat. Rev. Neurol. 12, 269–280. doi: 10.1038/nrneurol.2016.46 27063107

[B12] Fleissner C. K. Huebel N. Abd El-Bary M. M. Loh G. Klaus S. Blaut M. (2010). Absence of Intestinal Microbiota Does Not Protect Mice From Diet-Induced Obesity. Br. J. Nutr. 104, 919–929. doi: 10.1017/S0007114510001303 20441670

[B13] Furet J. P. Kong L. C. Tap J. Poitou C. Basdevant A. Bouillot J. L.. (2010). Differential Adaptation of Human Gut Microbiota to Bariatric Surgery-Induced Weight Loss: Links With Metabolic and Low-Grade Inflammation Markers. Diabetes 59, 3049–3057. doi: 10.2337/db10-0253 20876719 PMC2992765

[B14] Gozd-Barszczewska A. Koziol-Montewka M. Barszczewski P. Mlodzinska A. Huminska K. (2017). Gut Microbiome as a Biomarker of Cardiometabolic Disorders. Ann. Agric. Environ. Med. 24, 416–422. doi: 10.26444/aaem/75456 28954482

[B15] Jeon J. Lourenco J. Kaiser E. E. Waters E. S. Scheulin K. M. Fang X.. (2020). Dynamic Changes in the Gut Microbiome At the Acute Stage of Ischemic Stroke in a Pig Model. Front. Neurosci. 14, 587986. doi: 10.3389/fnins.2020.587986 33343283 PMC7744295

[B16] Kaakoush N. O. (2015). Insights Into the Role of Erysipelotrichaceae in the Human Host. Front. Cell Infect. Microbiol. 5, 84. doi: 10.3389/fcimb.2015.00084 26636046 PMC4653637

[B17] Karlsson F. H. Tremaroli V. Nookaew I. Bergstrom G. Behre C. J. Fagerberg B.. (2013). Gut Metagenome in European Women With Normal, Impaired and Diabetic Glucose Control. Nature 498, 99–103. doi: 10.1038/nature12198 23719380

[B18] Kasahara K. Krautkramer K. A. Org E. Romano K. A. Kerby R. L. Vivas E. I.. (2018). Interactions Between Roseburia Intestinalis and Diet Modulate Atherogenesis in a Murine Model. Nat. Microbiol. 3, 1461–1471. doi: 10.1038/s41564-018-0272-x 30397344 PMC6280189

[B19] Kasubuchi M. Hasegawa S. Hiramatsu T. Ichimura A. Kimura I. (2015). Dietary Gut Microbial Metabolites, Short-Chain Fatty Acids, and Host Metabolic Regulation. Nutrients 7, 2839–2849. doi: 10.3390/nu7042839 25875123 PMC4425176

[B20] Koliada A. Syzenko G. Moseiko V. Budovska L. Puchkov K. Perederiy V.. (2017). Association Between Body Mass Index and Firmicutes/Bacteroidetes Ratio in an Adult Ukrainian Population. BMC Microbiol. 17, 120. doi: 10.1186/s12866-017-1027-1 28532414 PMC5440985

[B21] Lee J. d’Aigle J. Atadja L. Quaicoe V. Honarpisheh P. Ganesh B. P.. (2020). Gut Microbiota-Derived Short-Chain Fatty Acids Promote Poststroke Recovery in Aged Mice. Circ. Res. 127, 453–465. doi: 10.1161/CIRCRESAHA.119.316448 32354259 PMC7415518

[B22] Ley R. E. Turnbaugh P. J. Klein S. Gordon J. I. (2006). Microbial Ecology: Human Gut Microbes Associated With Obesity. Nature 444, 1022–1023. doi: 10.1038/4441022a 17183309

[B23] Liu Y. Dai M. (2020). Trimethylamine N-Oxide Generated by the Gut Microbiota Is Associated With Vascular Inflammation: New Insights Into Atherosclerosis. Mediators Inflamm. 2020, 4634172. doi: 10.1155/2020/4634172 32148438 PMC7048942

[B24] Li N. Wang X. Sun C. Wu X. Lu M. Si Y.. (2019). Change of Intestinal Microbiota in Cerebral Ischemic Stroke Patients. BMC Microbiol. 19, 191. doi: 10.1186/s12866-019-1552-1 31426765 PMC6700817

[B25] Lozupone C. Knight R. (2005). UniFrac: A New Phylogenetic Method for Comparing Microbial Communities. Appl. Environ. Microbiol. 71, 8228–8235. doi: 10.1128/AEM.71.12.8228-8235.2005 16332807 PMC1317376

[B26] Luck H. Khan S. Kim J. H. Copeland J. K. Revelo X. S. Tsai S.. (2019). Gut-associated IgA(+) Immune Cells Regulate Obesity-Related Insulin Resistance. Nat. Commun. 10, 3650. doi: 10.1038/s41467-019-11370-y 31409776 PMC6692361

[B27] Marques C. Meireles M. Norberto S. Leite J. Freitas J. Pestana D.. (2016). High-Fat Diet-Induced Obesity Rat Model: A Comparison Between Wistar and Sprague-Dawley Rat. Adipocyte 5, 11–21. doi: 10.1080/21623945.2015.1061723 27144092 PMC4836488

[B28] Martin A. M. Yabut J. M. Choo J. M. Page A. J. Sun E. W. Jessup C. F.. (2019). The Gut Microbiome Regulates Host Glucose Homeostasis Via Peripheral Serotonin. Proc. Natl. Acad. Sci. U. S. A. 116, 19802–19804. doi: 10.1073/pnas.1909311116 31527237 PMC6778212

[B29] Meddings J. B. Swain M. G. (2000). Environmental Stress-Induced Gastrointestinal Permeability Is Mediated by Endogenous Glucocorticoids in the Rat. Gastroenterology 119, 1019–1028. doi: 10.1053/gast.2000.18152 11040188

[B30] Meng Q. Ma M. Zhang W. Bi Y. Cheng P. Yu X.. (2021). The Gut Microbiota During the Progression of Atherosclerosis in the Perimenopausal Period Shows Specific Compositional Changes and Significant Correlations With Circulating Lipid Metabolites. Gut Microbes 13, 1–27. doi: 10.1080/19490976.2021.1880220 PMC7954427 33691599

[B31] Morrison D. J. Preston T. (2016). Formation of Short Chain Fatty Acids by the Gut Microbiota and Their Impact on Human Metabolism. Gut Microbes 7, 189–200. doi: 10.1080/19490976.2015.1134082 26963409 PMC4939913

[B32] Okar S. V. Topcuoglu M. A. Yemisci M. Cakir Aktas C. Oguz K. K. Arsava E. M. (2020). Post-Stroke Inflammatory Response Is Linked to Volume Loss in the Contralateral Hemisphere. J. Neuroimmunol. 344, 577247. doi: 10.1016/j.jneuroim.2020.577247 32388192

[B33] Pan Y. Cai X. Jing J. Meng X. Li H. Wang Y.. (2017). Stress Hyperglycemia and Prognosis of Minor Ischemic Stroke and Transient Ischemic Attack: The CHANCE Study (Clopidogrel in High-Risk Patients With Acute Nondisabling Cerebrovascular Events). Stroke 48, 3006–3011. doi: 10.1161/STROKEAHA.117.019081 29051218

[B34] Park J. H. Ovbiagele B. (2016). Relationship of Functional Disability After a Recent Stroke With Recurrent Stroke Risk. Eur. J. Neurol. 23, 361–367. doi: 10.1111/ene.12837 26493027

[B35] Prame Kumar K. Wong C. H. (2020). Imbalance in the Force: The Dark Side of the Microbiota on Stroke Risk and Progression. Curr. Opin. Neurobiol. 62, 10–16. doi: 10.1016/j.conb.2019.10.002 31809996

[B36] Qin J. Li Y. Cai Z. Li S. Zhu J. Zhang F.. (2012). A Metagenome-Wide Association Study of Gut Microbiota in Type 2 Diabetes. Nature 490, 55–60. doi: 10.1038/nature11450 23023125

[B37] Roberts G. W. Quinn S. J. Valentine N. Alhawassi T. O’Dea H. Stranks S. N.. (2015). Relative Hyperglycemia, a Marker of Critical Illness: Introducing the Stress Hyperglycemia Ratio. J. Clin. Endocrinol. Metab. 100, 4490–4497. doi: 10.1210/jc.2015-2660 26485219

[B38] Rost N. S. Bottle A. Lee J. M. Randall M. Middleton S. Shaw L.. (2016). Stroke Severity is a Crucial Predictor of Outcome: An International Prospective Validation Study. J. Am. Heart Assoc. 5, e002433. doi: 10.1161/JAHA.115.002433 26796252 PMC4859362

[B39] Sadler R. Cramer J. V. Heindl S. Kostidis S. Betz D. Zuurbier K. R.. (2020). Short-Chain Fatty Acids Improve Poststroke Recovery Via Immunological Mechanisms. J. Neurosci. 40, 1162–1173. doi: 10.1523/JNEUROSCI.1359-19.2019 31889008 PMC6989004

[B40] Segata N. Izard J. Waldron L. Gevers D. Miropolsky L. Garrett W. S.. (2011). Metagenomic Biomarker Discovery and Explanation. Genome Biol. 12, R60. doi: 10.1186/gb-2011-12-6-r60 21702898 PMC3218848

[B41] Seo B. Jeon K. Moon S. Lee K. Kim W. K. Jeong H.. (2020). Roseburia Spp. Abundance Associates With Alcohol Consumption in Humans and Its Administration Ameliorates Alcoholic Fatty Liver in Mice. Cell Host Microbe 27, 25–40 e26. doi: 10.1016/j.chom.2019.11.001 31866426

[B42] Silveira-Nunes G. Durso D. F. Alves de Oliveira L. R. Jr Cunha E. H. M. Maioli T. U. Vieira A. T.. (2020). Hypertension Is Associated With Intestinal Microbiota Dysbiosis and Inflammation in a Brazilian Population. Front. Pharmacol. 11, 258. doi: 10.3389/fphar.2020.00258 32226382 PMC7080704

[B43] Spychala M. S. Venna V. R. Jandzinski M. Doran S. J. Durgan D. J. Ganesh B. P.. (2018). Age-Related Changes in the Gut Microbiota Influence Systemic Inflammation and Stroke Outcome. Ann. Neurol. 84, 23–36. doi: 10.1002/ana.25250 29733457 PMC6119509

[B44] Tan C. Wang H. Gao X. Xu R. Zeng X. Cui Z.. (2020). Dynamic Changes and Prognostic Value of Gut Microbiota-Dependent Trimethylamine-N-Oxide in Acute Ischemic Stroke. Front. Neurol. 11, 29. doi: 10.3389/fneur.2020.00029 32082246 PMC7005238

[B45] Tan C. Wu Q. Wang H. Gao X. Xu R. Cui Z.. (2021). Dysbiosis of Gut Microbiota and Short-Chain Fatty Acids in Acute Ischemic Stroke and the Subsequent Risk for Poor Functional Outcomes. JPEN J. Parenter. Enteral Nutr 45, 518–529. doi: 10.1002/jpen.1861 32473086 PMC8048557

[B46] Thaiss C. A. (2018). Microbiome Dynamics in Obesity. Science 362, 903–904. doi: 10.1126/science.aav6870 30467161

[B47] Turnbaugh P. J. Backhed F. Fulton L. Gordon J. I. (2008). Diet-Induced Obesity is Linked to Marked But Reversible Alterations in the Mouse Distal Gut Microbiome. Cell Host Microbe 3, 213–223. doi: 10.1016/j.chom.2008.02.015 18407065 PMC3687783

[B48] van den Munckhof I. C. L. Kurilshikov A. Ter Horst R. Riksen N. P. Joosten L. A. B. Zhernakova A.. (2018). Role of Gut Microbiota in Chronic Low-Grade Inflammation as Potential Driver for Atherosclerotic Cardiovascular Disease: A Systematic Review of Human Studies. Obes. Rev. 19, 1719–1734. doi: 10.1111/obr.12750 30144260

[B49] Vinolo M. A. Rodrigues H. G. Nachbar R. T. Curi R. (2011). Regulation of Inflammation by Short Chain Fatty Acids. Nutrients 3, 858–876. doi: 10.3390/nu3100858 22254083 PMC3257741

[B50] Vrieze A. Van Nood E. Holleman F. Salojarvi J. Kootte R. S. Bartelsman J. F.. (2012). Transfer of Intestinal Microbiota From Lean Donors Increases Insulin Sensitivity in Individuals With Metabolic Syndrome. Gastroenterology 143, 913–916 e917. doi: 10.1053/j.gastro.2012.06.031 22728514

[B51] Wang Y. Song F. Zhu J. Zhang S. Yang Y. Chen T.. (2017). GSA: Genome Sequence Archive. Genomics Proteomics Bioinf. 15, 14–18. doi: 10.1016/j.gpb.2017.01.001 PMC5339404 28387199

[B52] Wang Y. Wang Y. Zhao X. Liu L. Wang D. Wang C.. (2013). Clopidogrel With Aspirin in Acute Minor Stroke or Transient Ischemic Attack. N. Engl. J. Med. 369, 11–19. doi: 10.1056/NEJMoa1215340 23803136

[B53] Ward T. Larson J. Meulemans J. Hillmann B. Lynch J. Sidiropoulos D.. (2017) BugBase Predicts Organism Level Microbiome Phenotypes. bioRxiv [Preprint]. doi: 10 .1101/133462.

[B54] Wardlaw J. M. Smith E. E. Biessels G. J. Cordonnier C. Fazekas F. Frayne R.. (2013). Neuroimaging Standards for Research Into Small Vessel Disease and Its Contribution to Ageing and Neurodegeneration. Lancet Neurol. 12, 822–838. doi: 10.1016/S1474-4422(13)70124-8 23867200 PMC3714437

[B55] Wouters A. Nysten C. Thijs V. Lemmens R. (2018). Prediction of Outcome in Patients With Acute Ischemic Stroke Based on Initial Severity and Improvement in the First 24 H. Front. Neurol. 9, 308. doi: 10.3389/fneur.2018.00308 29867722 PMC5950843

[B56] Yang T. Santisteban M. M. Rodriguez V. Li E. Ahmari N. Carvajal J. M.. (2015). Gut Dysbiosis Is Linked to Hypertension. Hypertension 65, 1331–1340. doi: 10.1161/HYPERTENSIONAHA.115.05315 25870193 PMC4433416

[B57] Yin J. Liao S. X. He Y. Wang S. Xia G. H. Liu F. T.. (2015). Dysbiosis of Gut Microbiota With Reduced Trimethylamine-N-Oxide Level in Patients With Large-Artery Atherosclerotic Stroke or Transient Ischemic Attack. J. Am. Heart Assoc. 4, e00269. doi: 10.1161/JAHA.115.002699 PMC4845212 26597155

[B58] Zeng X. Gao X. Peng Y. Wu Q. Zhu J. Tan C.. (2019). Higher Risk of Stroke Is Correlated With Increased Opportunistic Pathogen Load and Reduced Levels of Butyrate-Producing Bacteria in the Gut. Front. Cell Infect. Microbiol. 9, 4. doi: 10.3389/fcimb.2019.00004 30778376 PMC6369648

