# Supplementary material for: Roseburia Abundance Associates With Severity, Evolution and Outcome of Acute Ischemic Stroke
Source: Front Cell Infect Microbiol. 2021 Oct 19;11:669322. doi: 10.3389/fcimb.2021.669322 (PMC8562073; doi:10.3389/fcimb.2021.669322)
Supplement: Supplementary file 1 [file DataSheet_1.docx]

**Supplementary Materials**

**Contents:**

**Table S1.** The top-50 common genera (sorted by the relative abundance).

**Figure S1.** Flow chart.

**Figure S2.** Comparison of gut microbiota diversity between minor stroke patients and non-minor stroke patients after PSM.

**Figure S3.** Bacteria with significant differences at the phylum level between minor stroke patients and non-minor stroke patients after PSM (Metastats analysis).

**Figure S4.** Significantly discriminative taxa between minor stroke patients and non-minor stroke patients after PSM.

**Figure S5.** Heatmap of Spearman correlation analysis between gut microbiota and biochemical parameters after PSM.

**Table S1.** The top-50 common genera (sorted by the relative abundance).

| 1. *Bacteroides* | 2. *Blautia* | 3. *Escherichia/Shigella* | 4. *Lachnospiracea incertae sedis* | 5. *Faecalibacterium* |
| --- | --- | --- | --- | --- |
| 6. *Unassigned* | 7. *Alistipes* | 8. *Enterococcus* | 9. *Streptococcus* | 10. *Ruminococcus* |
| 11. *Lactobacillus* | 12. *Parabacteroides* | 13. *Klebsiella* | 14. *Bifidobacterium* | 15. *Prevotella* |
| 16. *Ruminococcus2* | 17. *Dorea* | 18. *Akkermansia* | 19. *Collinsella* | 20. *Clostridium XVIII* |
| 21. *Oscillibacter* | 22. *Phascolarctobacterium* | 23. *Clostridium XlVa* | 24. *Coprococcus* | 25. *Roseburia* |
| 26. *Megamonas* | 27. *Anaerostipes* | 28. *Megasphaera* | 29. *Gemmiger* | 30. *Dialister* |
| 31. *Fusicatenibacter* | 32. *Clostridium_IV* | 33. *Barnesiella* | 34. *Holdemanella* | 35. *Veillonella* |
| 36. *Anaerobacterium* | 37. *Fusobacterium* | 38. *Butyricicoccus* | 39. *Romboutsia* | 40. *Flavonifractor* |
| 41. *Butyricimonas* | 42. *Clostridium XlVb* | 43. *Clostridium sensu stricto* | 44. *Parasutterella* | 45. *Christensenella* |
| 46. *Hungatella* | 47. *Paraprevotella* | 48. *Erysipelotrichaceae incertae sedis* | 49. *Catenibacterium* | 50. *Odoribacter* |

**Figure S1. Flow chart.**

**
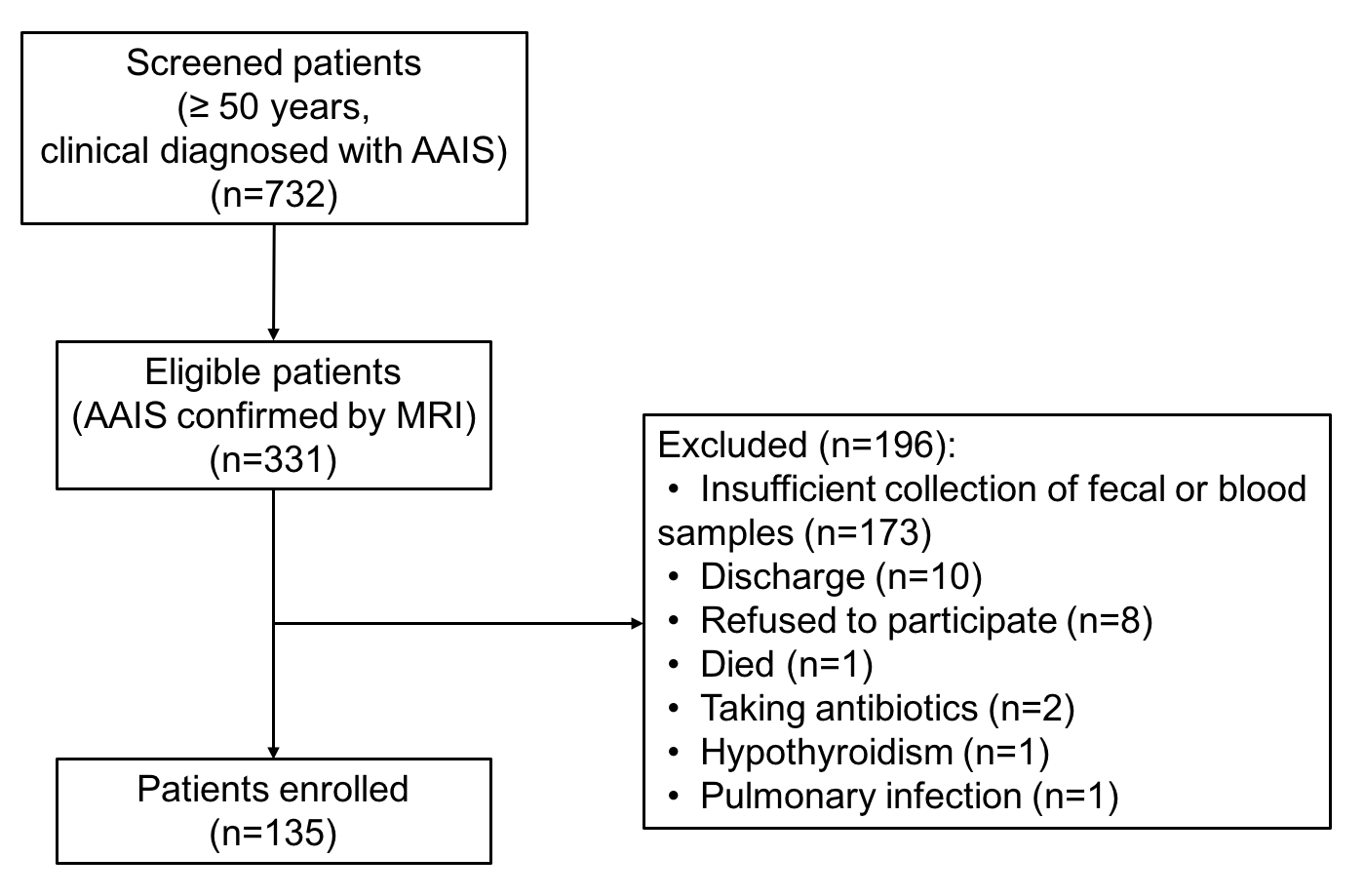
**

Abbreviations: AAIS = acute anterior ischemic stroke; MRI = Magnetic Resonance Imaging.

**Figure S2.** Comparison of gut microbiota diversity between minor stroke patients and non-minor stroke patients after PSM.

**
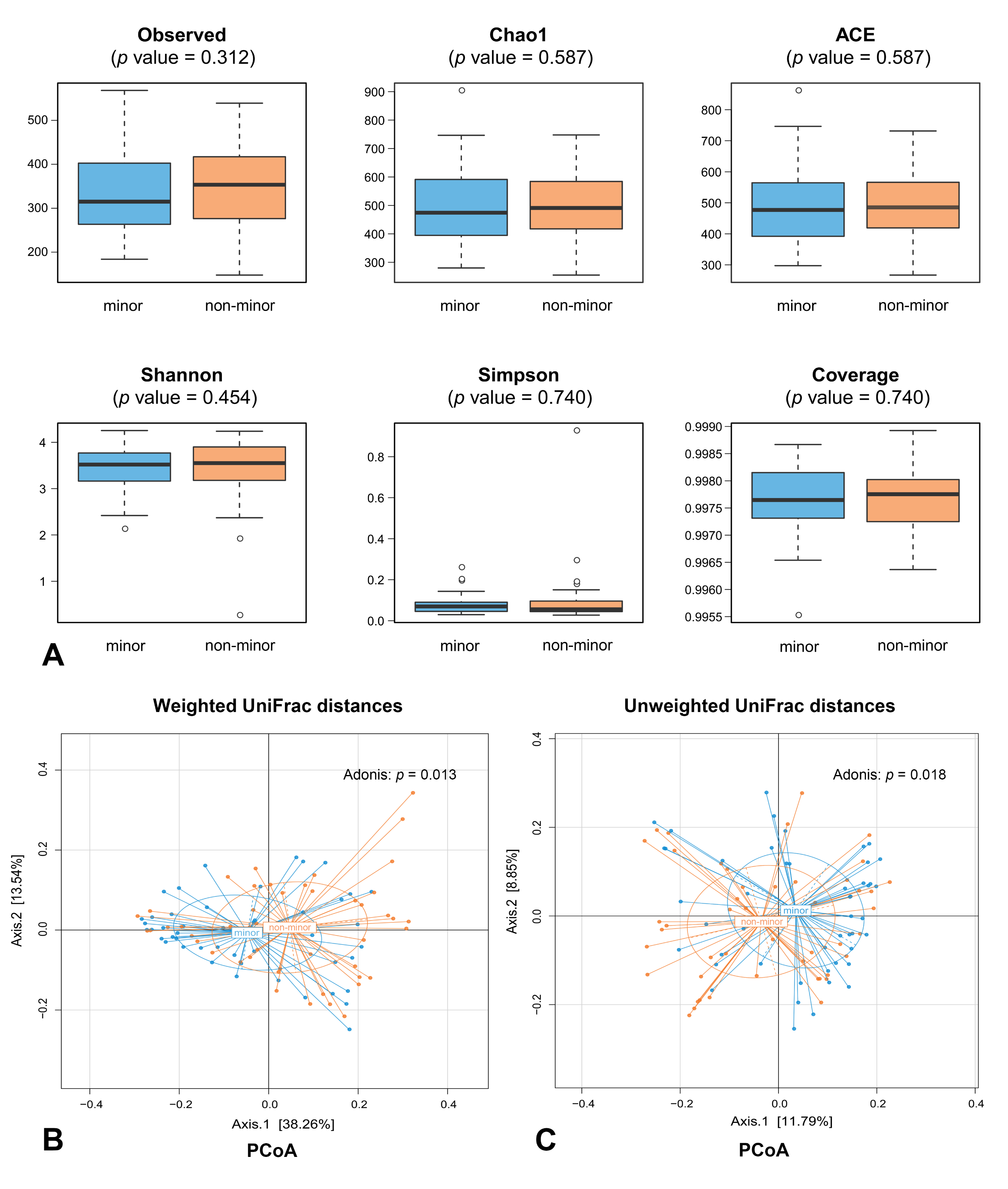
**

(A) Within-individual (α) diversity including observed species, Chao 1 and ACE, Shannon index, Simpson index and coverage index in minor stroke patients (blue) and non-minor stroke patients (orange). Boxes represent the interquartile ranges, lines inside the boxes denote medians, and circles are outliers. Between-individual (β) diversity, including principal coordinate analysis (PCoA) based on weighted UniFrac distances (B) and unweighted UniFrac distances (C), was tested by Adonis. The blue circles represent samples of minor stroke patients and orange circles represent samples of non-minor stroke patients.

Abbreviations: PSM = propensity score-matched analysis; PCoA = principle coordinate analysis.

**Figure S3.** Bacteria with significant differences at the phylum level between minor stroke patients and non-minor stroke patients after PSM (Metastats analysis).


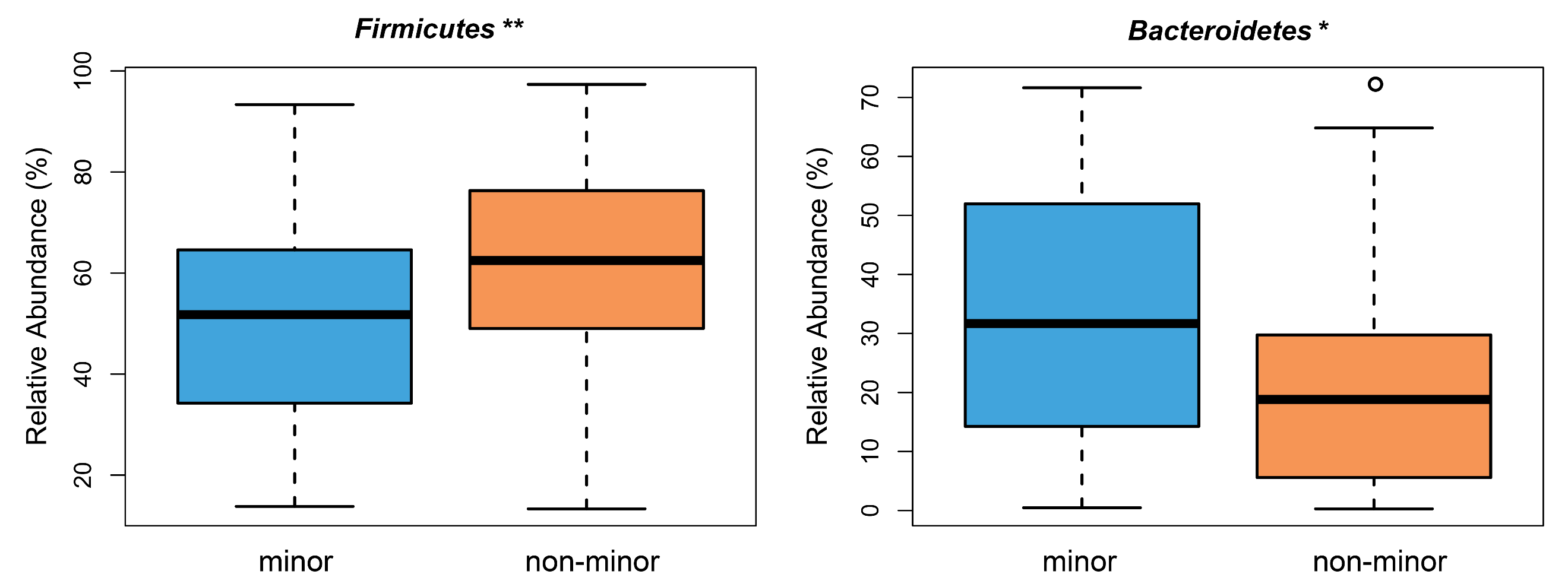


Boxes represent the interquartile ranges, lines inside the boxes denote medians, and circles are outliers. *p < 0.05, **p < 0.01.

**Figure S4.** Significantly discriminative taxa between minor stroke patients and non-minor stroke patients after PSM.


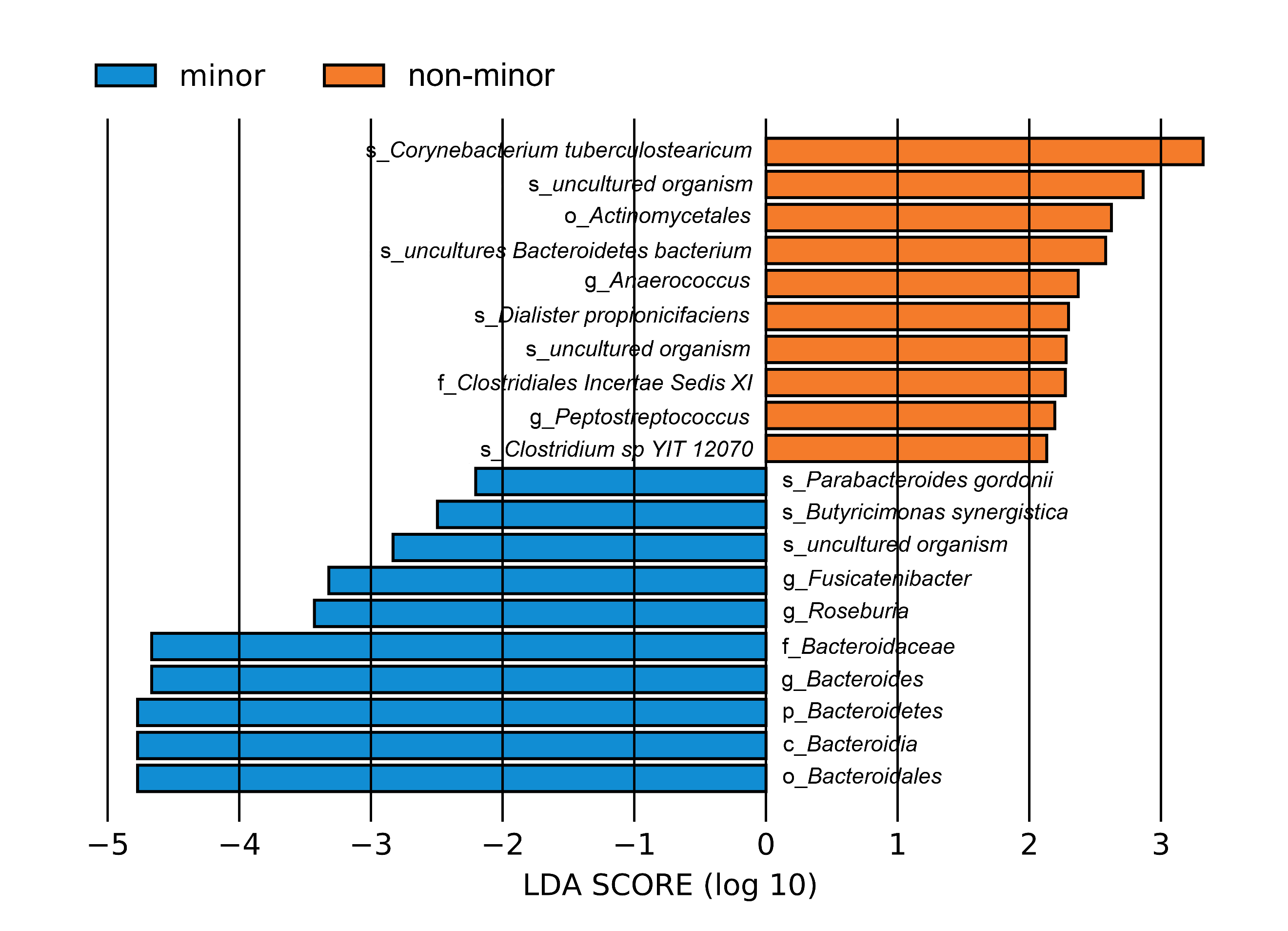


Abbreviations: PSM = propensity score-matched analysis.

**Figure S5.** Heatmap of Spearman correlation analysis between gut microbiota and biochemical parameters after PSM.

**
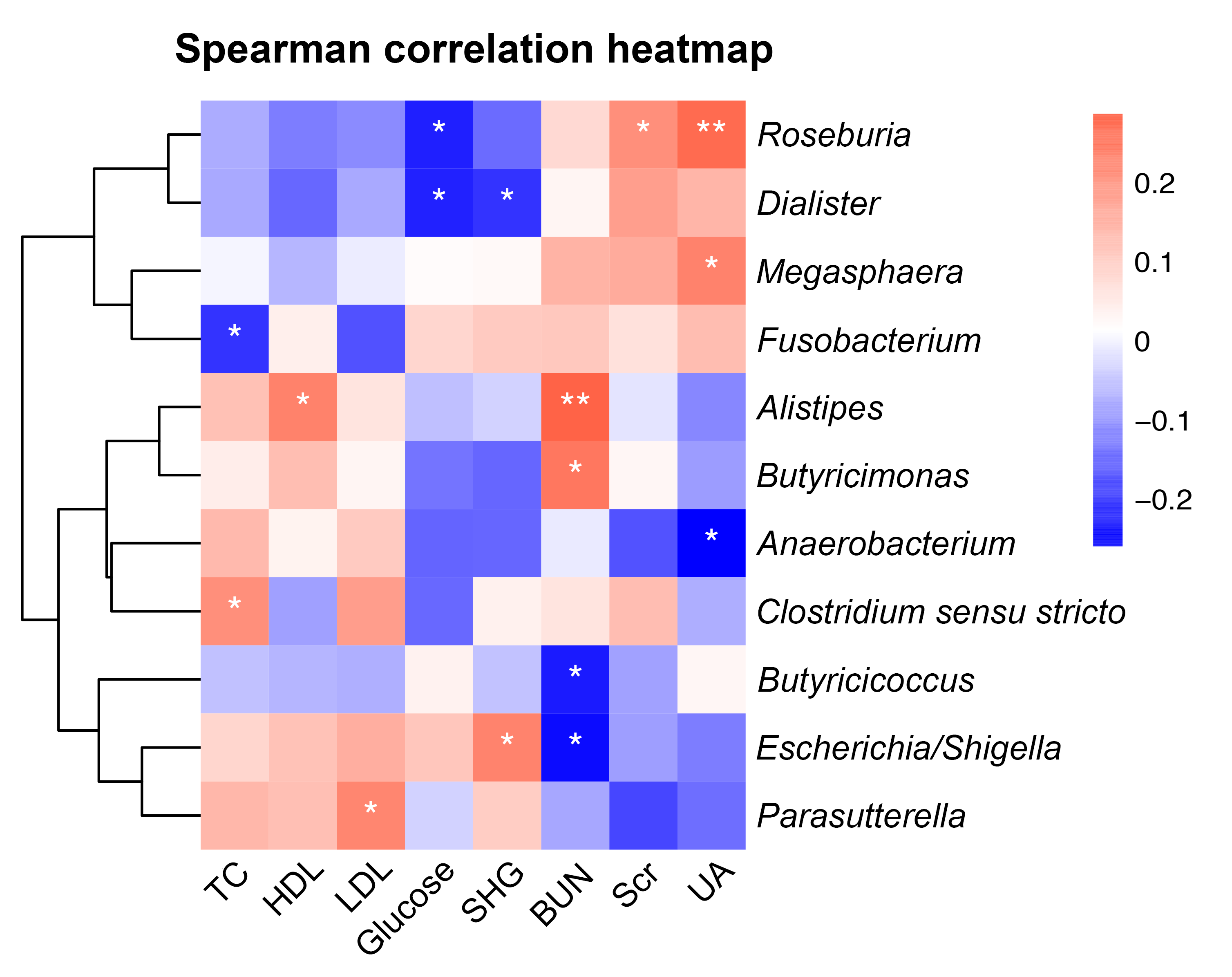
**

*p < 0.05, **p < 0.01.

Abbreviations: PSM = propensity score-matched analysis; Abbreviations: TC = total cholesterol; HDL = high-density lipoprotein cholesterol; LDL = low-density lipoprotein cholesterol; SHG = stress hyperglycemia; BUN = blood urea nitrogen; Scr = serum creatinine; UA = uric acid.
